# Surface accumulation and acid–base equilibrium of phenol at the liquid–vapor interface†

**DOI:** 10.1039/d4cp02212b

**Published:** 2024-08-23

**Authors:** Clemens Richter, Rémi Dupuy, Florian Trinter, Tillmann Buttersack, Louisa Cablitz, Shirin Gholami, Dominik Stemer, Christophe Nicolas, Robert Seidel, Bernd Winter, Hendrik Bluhm

**Affiliations:** a Fritz Haber Institute of the Max Planck Society Faradayweg 4–6 14195 Berlin Germany bluhm@fhi-berlin.mpg.de crichter@fhi-berlin.mpg.de; b CNRS, Laboratoire de Chimie Physique – Matière et Rayonnement, Sorbonne Université F-75005 Paris Cedex 05 France; c Synchrotron SOLEIL, L’Orme des Merisiers Saint-Aubin – BP 48 91192 Gif-sur-Yvette Cedex France; d Helmholtz-Zentrum Berlin für Materialien und Energie Hahn-Meitner-Platz 1 14109 Berlin Germany

## Abstract

We have investigated the surfactant properties of phenol in aqueous solution as a function of pH and bulk concentration using liquid-jet photoelectron spectroscopy (LJ-PES) and surface tension measurements. The emphasis of this work is on the determination of the Gibbs free energy of adsorption and surface excess of phenol and its conjugate base phenolate at the bulk p*K*_a_ (9.99), which can be determined for each species using photoelectron spectroscopy. These values are compared to those obtained in measurements well below and well above the p*K*_a_, where pure phenol or phenolate, respectively, are the dominant species, and where the Gibbs free energy of adsorption determined from surface tension and LJ-PES data are in excellent agreement. At the bulk p*K*_a_ the surface-sensitive LJ-PES measurements show a deviation of the expected phenol/phenolate ratio in favor of phenol, *i.e.*, an apparent upward shift of the 
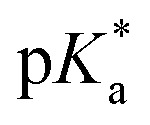
 at the surface. In addition, the Gibbs free energies of adsorption determined by LJ-PES at the bulk p*K*_a_ for phenol and phenolate deviate from those observed for the pure solutions. We discuss these observations in view of the different surface propensity of phenol and phenolate as well as potential cooperative interactions between them in the near-surface region.

## Abstract 2

We have investigated the surfactant properties of phenol in aqueous solution as a function of pH and bulk concentration using liquid-jet photoelectron spectroscopy (LJ-PES) and surface tension measurements.

## Graphical abstract

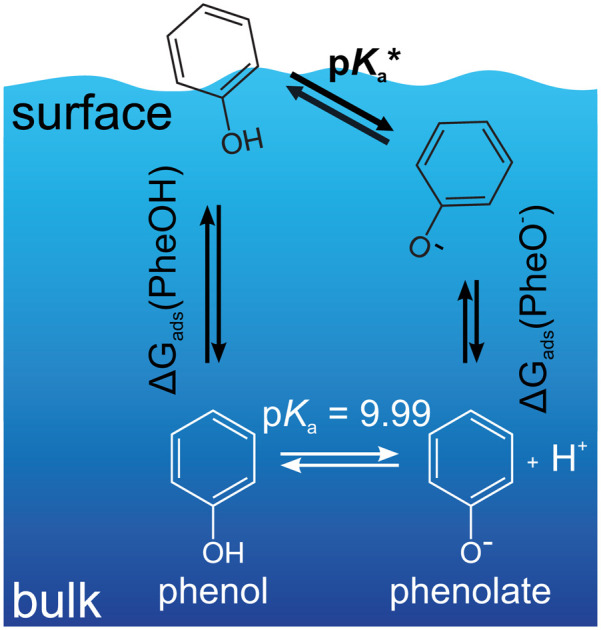

## 1 Introduction

The air–water interface is omnipresent in nature, *e.g.*, in marine and freshwater environments, atmospheric aqueous aerosols, as well as fog and cloud droplets.^1^ It is involved in numerous heterogeneous chemical reactions of environmental and atmospheric importance and may show drastically increased reactivity compared to reactions in the bulk solution.^2^ Under realistic environmental conditions liquid–vapor interfaces are covered by various kinds of amphiphilic organic compounds, consisting of a polar hydrophilic and a non-polar hydrophobic part. These organic molecules typically alter surface properties, like the surface tension^3^ or the evaporation rate.^4^ Therefore, a fundamental understanding of heterogeneous processes at liquid–vapor interfaces requires detailed knowledge of the propensity and properties of surfactants at aqueous solution–vapor interfaces.

In this study we focus on the interfacial properties of aqueous phenol solutions. The phenolic structure motif is abundant in organic matter, *e.g.*, in plants, and phenol and related phenolic compounds are common natural and anthropogenic pollutants, found in the form of aerosols or as a component of wastewater. Specifically, the oxidation of phenol and its derivatives with ozone is studied intensely in the context of ageing of organic aerosols and wastewater treatment.^5–8^ As an amphiphilic organic compound with a hydroxyl group and an aromatic C_6_ ring, phenol naturally exhibits a relatively high preference for accumulating at the aqueous interface.

Early experiments on this system were concerned with the surface tension of neutral phenol solutions as a function of temperature and bulk concentration.^9,10^ More recent spectroscopic investigations on phenol aqueous solutions focused on the impact of the aqueous environment on its outer-valence electronic structure, using computations and photoelectron spectroscopy.^11–13^ In non-linear optical second-harmonic generation (SHG) and sum-frequency generation (SFG) studies the average molecular orientation of phenol at the liquid–vapor interface was determined.^14,15^ Time-resolved SFG experiments found a significantly enhanced (photo-)reactivity of phenol at the solution–air interface, with surface reaction rates exceeding those in the bulk by a factor of about 10^4^.^16^

One intriguing question concerning aqueous solution–vapor interfaces is whether acid–base equilibria differ from that in the bulk, and whether this can be attributed to a different local pH, a different p*K*_a_ at the interface or by different surface propensities/chemical activities, or a combination of these factors. The apparent surface 
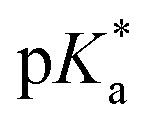
 of aqueous phenol solutions was investigated using SFG on extended solution–vapor interfaces^17^ and UV photoemission on nanoaerosols.^12^ In both studies neutral phenol was detected in the interface region even around pH 12,^12,17^ where it is expected that the equilibrium is strongly shifted towards phenolate (99% at pH 12).

**Fig. 1:**
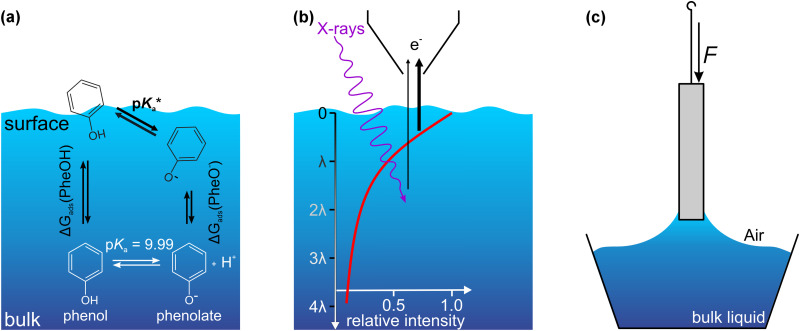
(a) Equilibria between phenol and phenolate at the surface and in the bulk of a phenol solution. The propensity of phenol and phenolate for the surface is described by their respective Gibbs adsorption enthalpy. Due to the less favorable solvation of phenol (compared to the charged phenolate) it is expected that is has a higher surface propensity. While the concentration of phenol and phenolate in the bulk is equal at the p*K*_a_, their concentration is different in the few-nm-thick interface region due to the difference in solvation, giving rise to an apparent shift in the “surface 
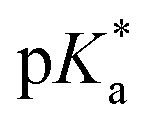
” *versus* that in the bulk. (b) Photoelectron spectroscopy has tunable surface-sensitivity, with a probing depth which is determined by the electron inelastic mean free path *λ*. In the experiments presented here *λ* is on the order of 2 nm, *i.e.*, about 65% of the signal originates from this depth. (c) A surface tension measurement using the Du Noüy–Padday method determines the force acting on a probe that is extracted from the solution. This macroscopic method thus averages over all forces and species that contribute to the phenomenon of surface tension. Please note that the length scales in panels (b) and (c) are very different (nm *versus* mm, respectively).

Here, we expand on these previous studies and investigate the adsorption enthalpies and surface concentrations of phenol and phenolate over a wide pH range, with focus on the adsorption characteristics of phenol and phenolate at pH values around the bulk solution p*K*_a_ of 9.99 [see Fig. 1(a)].

The experiments were performed by a combination of liquid-jet photoelectron spectroscopy (LJ-PES) with an electron inelastic mean free path of about 2 nm into solution^18,19^ [Fig. 1(b)], and surface tension measurements, which provide an indirect probe of the concentration of solute species at the interface [Fig. 1(c)]. While Tamburello-Luca *et al.*^14^ and Rao *et al.*^17^ supplemented their spectroscopic investigations with surface tension measurements to estimate the surface coverage, a systematic combination of the information obtained by spectroscopic measurements and surface tension measurements has not yet been attempted for the phenol–water system, to the best of our knowledge. Only a few studies correlated the results of surface tension measurements, in particular the solute surface excess concentration, to the photoelectron signal intensity of other organic solutes.^20–22^

In the investigations described in the following we have first compared the surface excess and surface adsorption enthalpies for pure phenol and phenolate solutions as determined by LJ-PES and surface tension measurements as a function of bulk concentration. We found a remarkably good agreement of the values obtained by these conceptually very different methods. We have then used the chemical sensitivity of LJ-PES, which is able to resolve phenol and phenolate species in valence and core-level photoemission spectra, to determine the surface excess and adsorption enthalpy for each species at the bulk solution p*K*_a_, where both species are present side-by-side in the solution. These species-resolved values are then compared to the global value that is obtained by surface tension measurements, which averages over the contribution of phenol and phenolate to the surface tension. The LJ-PES data show that an equal concentration of phenol and phenolate, which is characteristic for the p*K*_a_, is found in the interface region at a higher pH value (11.1(3)) than in the bulk (9.99), an observation that can be explained by the higher propensity of the uncharged phenol for the interface.

## 2 Methods

### 2.1 Photoelectron spectroscopy

We performed ultraviolet photoelectron spectroscopy (UPS) and X-ray photoelectron spectrocopy (XPS) experiments on liquid microjets (LJs). UPS experiments were carried out using the EASI (electronic structure of aqueous solutions and interfaces) setup dedicated to LJ-PES investigations.^23^ In brief, the setup consists of a differentially pumped hemispherical electron analyzer (Scienta Omicron HiPP-3) attached to an interaction chamber housing a cylindrical LJ. A monochromatized helium plasma-discharge UV light source was used with He-II α radiation (40.814 eV). The LJ had a diameter of 28 μm and a velocity of ∼20 ms^−1^. The liquid freezes after few centimeters travel due to evaporative cooling. The ice is then collected at the wall of a cold trap containing liquid nitrogen (LN_2_) to maintain a base pressure of 10^−5^–10^−4^ mbar. The focal size of the VUV light in the interaction region is on the order of 300 × 300 μm^2^, *i.e.*, significantly exceeding the size of the liquid microjet.

XPS experiments were performed using the LJ module of the PLEIADES beamline of the SOLEIL synchrotron facility (Paris, France).^24^ The LJ module is attached to the experimental chamber equipped with a hemispherical electron analyzer (VG Scienta R4000). It consists of a differentially pumped rectangular volume housing a glass capillary of 45 μm diameter from which the LJ is ejected using an HPLC pump (jet velocity ∼20 ms^−1^) and collected through a heated catcher. This compartment has orifices in the direction orthogonal to the liquid-jet propagation axis to enable intersection of the jet by the incident synchtrotron radiation and the detection of photoelectrons. It is placed in front of the entrance of the electron analyzer. The PLEIADES beamline offers fully polarizable synchrotron radiation over a range of 50–1000 eV with a focal size of 50 × 120 μm^2^ (*v* × *h*). During the measurements the pressure was maintained in the 10^−3^ mbar range in the jet housing, and in the 10^−6^ mbar range in the differentially pumped spectrometer chamber. For the XPS experiments a photon energy range of 100–640 eV was used with an analyzer pass energy of 50 eV, providing an energy resolution of about 50 meV. A few data points were recorded with the SOL^3^PES setup at the UE52_SGM beamline at the synchrotron facility BESSY II (Berlin, Germany). The experimental setup is similar to the one described above; details are decribed elsewhere.^25^

All solutions were prepared with MilliQ water and added 50 mM NaCl to ensure sufficient electrical conductivity in the photoemission experiments. Phenol (>99%, Sigma-Aldrich and Carl Roth) was added as received to obtain aqueous solutions with the desired concentration. For pH adjustments NaOH was used, and the solution pH was measured with a pH meter (VWR pHenomenal pH 1100 L).

### 2.2 UPS/XPS data analysis

The spectra were fitted using the KolXPD software package (Kolibrik.net, Czech Republic). For all spectra a linear background was employed. Condensed-phase peaks were fitted with Gaussian and gas-phase peaks with Voigt profiles.

The analysis of valence electron spectra started with the description of pure water. Subsequently, phenol solution spectra were fitted using the water valence structure as a background, fixing the relative positions of the gas-phase water peaks and the relative positions and peak widths of the liquid-water peaks. For the phenol signal three additional peaks were introduced, one for the HOMO and another for the HOMO−1 in accordance with ref. 11 and 26. The third peak was used to capture phenol-induced changes in the region overlapping with the liquid-water 1b_1_ signal.

C 1s core-level (XPS) spectra of phenol solutions were fitted using two Gaussian peaks for CH_*x*_ and COH (or CO^−^). The oxygen signal of neutral phenol was obscured by the much stronger contribution from water in the O 1s spectra. The O 1s signal of negatively charged phenolate can be observed at high concentrations due to the chemical shift of the CO^−^ O 1s peak to a lower binding energy compared to neutral COH. Exemplary spectra and a more detailed description of the fitting routine and the utilized constraints specifically for solutions including phenol and phenolate are shown in the ESI† (see Fig. S1, S3, and S4).

### 2.3 Surface tension measurements

Surface tension measurements of phenol and sodium phenolate solutions were performed to determine their respective surface excess concentration over the bulk concentration ranges covered in the UPS and XPS measurements. The surface tension was measured using the EZ-Pi+ tensiometer (Kibron Inc, Helsinki). The measurements are based on the Du Noüy–Padday method^27^ using a metal-alloy probe (DyneProbe, Kibron Inc, Helsinki) which was routinely flamed before each measurement for cleaning and to ensure consistent wetting by the solutions.

## 3 Results and discussion

**Fig. 2:**
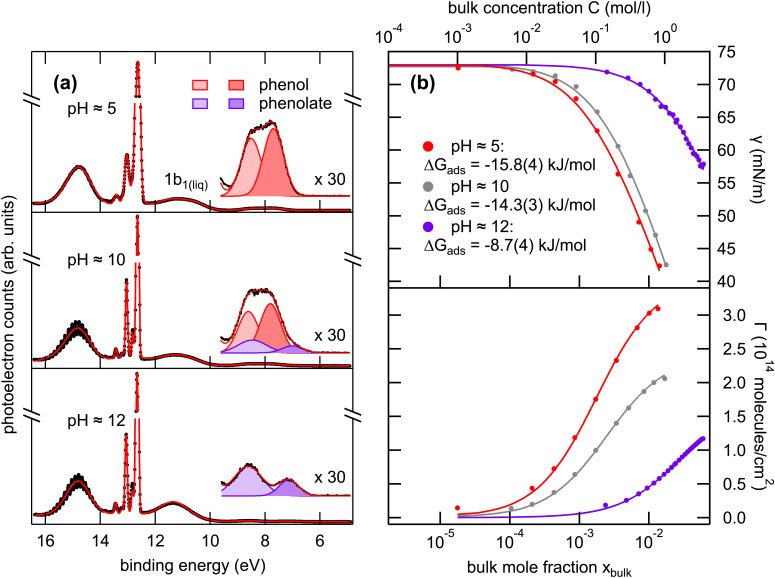
(a) Valence electron spectra of 800 mM phenol solutions at pH 5, 10 (the bulk p*K*_a_), and 12, recorded with *hν* = 40.814 eV (He II α). The inset shows an enlarged view of the HOMO and HOMO−1 states of phenol. (b) Surface tension *γ* (upper panel) and surface concentration *Γ* (lower panel) of phenol at pH 5, 10, and 12 as a function of the bulk concentration.

We systematically investigated phenol solutions over a large concentration range at pH 5, 10, and 12 using LJ-PES and surface tension measurements. Representative data are compiled in Fig. 2. In Fig. 2(a), the valence electron spectra of 800 mM phenol solutions at pH 5, 10, and 12 area are displayed, with the binding energy scale referenced to the position of the liquid water 1b_1_ valence level binding energy of 11.33 eV.^28^ In this study, we did not attempt to determine the absolute binding energies using the procedures detailed in ref. 28–30, since the absolute binding energy is not essential for the characterization of the surface propensity of phenol. The valence electron spectra are dominated by the signature of gaseous and liquid water. The high gas-phase intensity (sharp features around 13 eV) in the spectra is caused by the relatively large spot size of the UV radiation which significantly exceeds the dimensions of the liquid jet filament, and thus a large portion of the gas phase around the liquid jet is detected.

The outer valence states of phenol at binding energies between 7 to 10 eV are, however, well separated from the liquid water bands. This spectral region is enlarged in the insets in Fig. 2(a). The two contributions are assigned to the HOMO and HOMO−1 orbitals of phenol.^11^ Depending on the solution pH the HOMO and HOMO−1 signatures change significantly and therefore allow to determine the protonation state of the solute. At pH ∼ 5 the neutral phenol is the dominating solute species, while at pH ∼ 12 the negatively charged phenolate is the predominant species. At the bulk p*K*_a_ of pH 10, both species are simultaneously present in the spectra.

### 3.1 Determination of surface excess and adsorption energies

We first take a closer look at the results of the surface tension measurements in Fig. 2(b). Displayed are the surface tension curves of phenol solutions as a function of the bulk concentration for the pH values of 5, 10 (p*K*_a_), and 12. The upper panel displays the experimentally determined surface tension *γ* of phenol (red symbols) and phenolate (violet symbols) as a function of their bulk mole fraction *χ*_bulk_ (bottom axis) and bulk concentration *C* (top axis). The data were fitted using the Szyszkowski–Langmuir equation (eqn (1)) to determine the Gibbs free energy of adsorption of phenol/phenolate at the water surface:^31^1
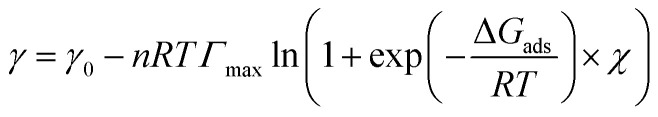


Here, *γ* is the experimental surface tension of the solution (in mN m^−1^), *γ*_0_ the surface tension of the pure solvent, here water (72.8 mN m^−1^ (ref. 32)), *R* the universal gas constant (in J K^−1^ mol^−1^), *T* the absolute temperature (295 K), *Γ*_max_ the maximum surface concentration of the solute (in mol m^−2^), Δ*G*_ads_ the Gibbs free energy of adsorption (in J mol^−1^), and *χ* the mole fraction of the solute. The additional factor *n* is a constant which depends on the charge of the solute species and therefore on the effects of counter ions in the solution. For neutral molecules *n* = 1 and for ionic species *n* = 2. For phenol at pH 5 we obtain a Δ*G*_ads_ of −15.8(4) kJ mol^−1^ and for phenolate at pH 12 −8.7(4) kJ mol^−1^, indicating a higher surface affinity for the undissociated phenol species. For the pH 10 solution, *i.e.*, at the bulk p*K*_a_, we determined a Δ*G*_ads_ of −14.3(3) kJ mol^−1^. Here, a value of *n* = 1.5 was used, assuming an average charge due to the presence of neutral and ionic surfactants.

The lower panel in Fig. 2(b) shows the corresponding surface concentrations *Γ* estimated from the surface tension data. The respective surface excess concentrations *Γ* (in molecules cm^−2^) were estimated using the Gibbs adsorption equation (eqn (2)):^31,33^2
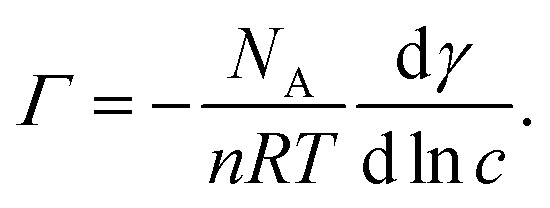


This equation relates *Γ* to the derivative of *γ* with respect to the logarithm of the solute concentration ln *c*. The lower panel of Fig. 2(b) shows the estimated surface concentrations of phenol at pH 5 and 10 and phenolate at pH 12 as a function of their bulk mole fraction and bulk concentration. To make it more illustrative the quantity *Γ* can be expressed in terms of a surface fraction *f*_s_ occupied by phenol and phenolate at the solution surface. Based on the simplified assumption that complete coverage of the liquid–vapor interface leads to the formation of a phenol phase with a density equal to bulk phenol, one can derive the maximum surface fraction of phenol *f*_phenol_ from the determined surface concentrations, thus giving a value for the maximum molecular surface coverage. For the phenol solutions at pH 5, 10, and 12 at 800 mM bulk concentration the values for *f*_s,min_ are 0.87, 0.57, and 0.16, respectively. Details on the calculation of the surface coverage are given in the ESI.†

### 3.2 UPS of pure phenol (pH 5) and phenolate (pH 12) solutions

**Fig. 3:**
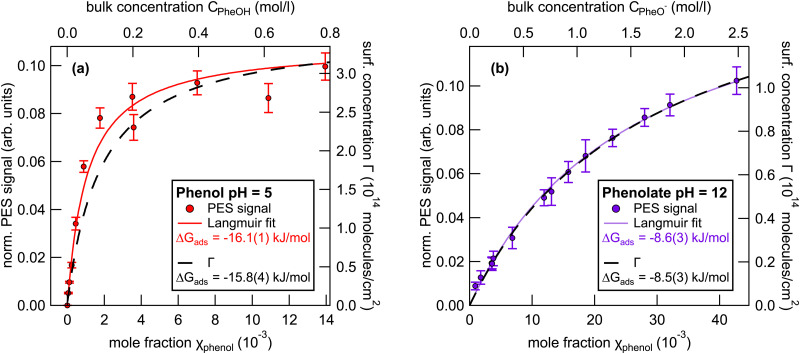
Left axes: Integrated (a) phenol and (b) phenolate HOMO+HOMO−1 peak intensity as a function of the mole fraction *χ* (bottom) and the bulk concentration *C* (top). Right axes: Surface excess concentration *Γ* estimated from the corresponding surface tension measurements. The error bars represent the standard deviation of the phenol valence signal due to stochastic errors in the measurements.

We now focus on the results of the LJ-PES measurements, starting with the measurements of neutral phenol, at pH 5, as a function of the bulk concentration. Photoelectron spectra of 1 to 800 mM phenol solutions were recorded to determine the phenol HOMO and HOMO−1 signal intensities. Fig. 3(a) shows the integrated phenol valence signal as a function of the mole fraction *χ*_phenol_ (bottom axis) and the respective bulk concentration *C*_phenol_ (top axis).

The signal was normalized to the signal intensity of the liquid-water 1b_1_ peak of a reference spectrum of pure water. This normalization is used to account for potential fluctuations in the detection efficiency in the experiments. The integrated peak intensity *versus* bulk mole fraction data were fitted using a Langmuir adsorption isotherm model:^34^3
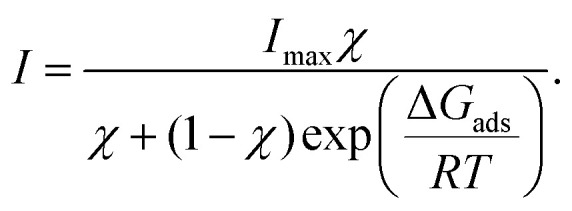


Here, *I* is the integrated photoelectron signal, *I*_max_ is the maximum intensity at monolayer saturation coverage, and *χ* is the bulk mole fraction. From this fit a Gibbs free energy of adsorption of −16.1(1) kJ mol^−1^ was determined. In addition, Fig. 3(a) also shows the surface concentration *Γ* (black line, right axis) which was estimated from surface tension measurements using eqn (2). The Langmuir fit to the UPS data agrees well with the surface excess concentration determined from surface tension measurements.

The same analytical approach was applied to the phenolate valence spectra at pH 12 (see Fig. 2). Fig. 3(b) shows the integrated HOMO + HOMO−1 intensity of phenolate normalized to the liquid-water 1b_1_ intensity of pure water as a function of the bulk concentration.

The fit to the UPS data using the Langmuir adsorption model is shown as a violet trace. The estimated surface concentration based on these data is shown as black trace, plotted against the right axis. The free energy of adsorption determined from the electron spectra is −8.6(3) kJ mol^−1^, compared to −8.5(3) kJ mol^−1^ as determined from the surface tension measurements, *i.e.*, the two methods yield adsorption free energies that are in remarkably good agreement.

To corroborate the results on surface adsorption of phenol and phenolate obtained from the valence electron spectra, we performed analogous XPS measurements of phenol solutions. The resulting free adsorption energies of these measurements agree well with the ones obtained from valence electron spectra. All results are summarized in Table 1 below. The details of the XPS measurements and representative spectra are given in the ESI† (see Fig. S4).

**Table 1:** Compilation of Gibbs free energies of adsorption Δ*G*_ads_ for phenol/phenolate aqueous solutions determined with UPS, XPS, and surface tension measurements. All values are given in kJ mol^−1^

Species	pH	Δ*G*^UPS^_ads_	Δ*G*^XPS^_ads_	Δ*G*^ST^_ads_
Phenol	5	−16.1(1)	−16.7(3)	−15.8(4)
Phenolate	12	−8.6(3)	−8.1(2)	−8.5(3)
Phenol	10	−14.0(3)	−12.7(6)	−14.3(3)
Phenolate	10	−14.5(4)	−13.7(4)	

### 3.3 Acid–base equilibrium at the solution–vapor interface

After discussing the results of the pure phenol and phenolate solutions in the previous section, we now turn our attention to the pH region around the bulk p*K*_a_, where both species coexist. Their relative abundance in the bulk as a function of pH is described by the acid–base equilibrium [see Fig. 1(a)] of weak acids and bases in the form of the Henderson–Hasselbalch equation:4
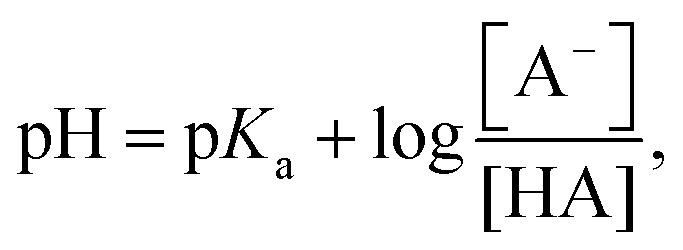
with p*K*_a_ as the negative logarithm of the acid dissociation constant 
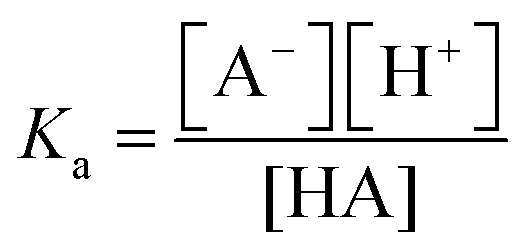
. We note that this equation assumes that the activity coefficients are 1 for all solutes.

This equation is often expressed in a more convenient form which describes the pH-dependent fraction of one species relative to the sum of both components. For instance, the acid fraction *f*_a_ as a function of the solution pH is given by:5
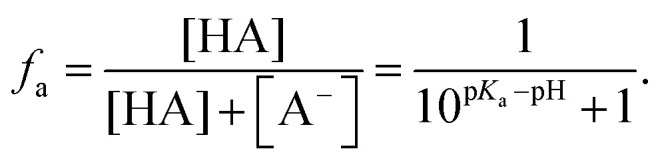


In the present case, phenol represents the weak acid and phenolate its conjugated base, with a p*K*_a_ of 9.99 for the bulk solution.^35^

Previous investigations have shown deviations from the bulk behavior specifically in the vicinity of interfaces, such as liquid–vapor or liquid–solid interfaces.^36–41^ We here investigate the acid–base equilibrium of phenol and phenolate at the liquid–vapor interface, utilizing the inherent surface sensitivity of photoelectron spectroscopy due to the short inelastic mean free path of the electrons, estimated to be on the order of 2 nm in our UPS measurements, based on the kinetic energy of the photoelectrons and estimates of the effective attenuation length in liquid water.^18,19^

Valence electron spectra were recorded from 200 mM phenol solutions at pH values in the range from 5 to 13. The spectra were fitted using constraints for the peak parameters obtained from the single-component spectra of phenol (pH 5) and phenolate (pH 12). The acid fraction *f*_a_ was determined for each spectrum from the integrated HOMO and HOMO−1 signatures of the two species in accordance with eqn (5).

**Fig. 4:**
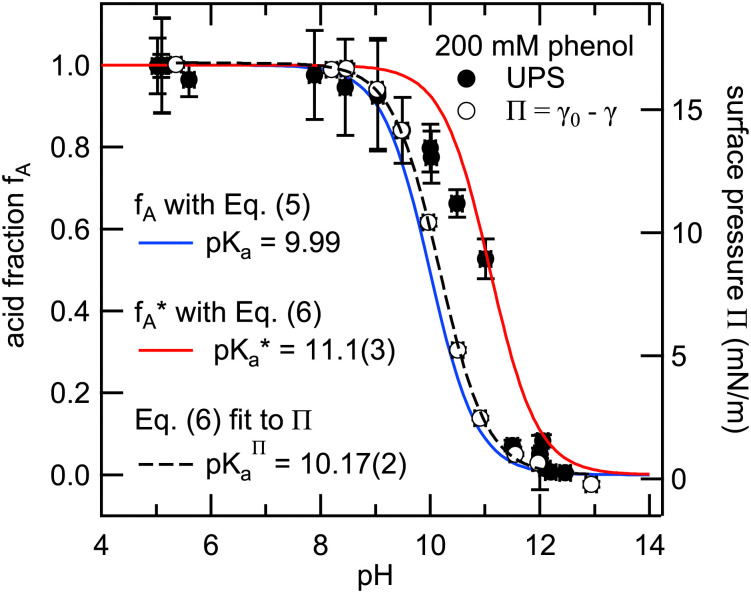
Left axis: Experimentally determined phenol acid fractions *f*_a_ (solid symbols) as a function of the solution pH for 200 mM phenol solutions. The blue trace represents the bulk acid fraction, as determined from the Henderson–Hasselbalch equation with p*K*_a_ = 9.99. The red trace is the modified acid fraction, taking into account the different surface concentrations of phenol and phenolate with p*K*_a_ − log(*g*) (*g* = *Γ*_PheOH_/*Γ*_PheO^−^_ = 12.6 at 200 mM bulk concentration). Right axis: Surface pressure *Π* (open symbols) as a function of the solution pH, as determined from surface tension measurements.

Fig. 4 shows the experimentally determined acid fractions (black symbols) from the valence electron spectra of 200 mM phenol solutions as a function of the solution pH. A comparison of the experimental data with the modeled data (blue trace) based on eqn (5) using the tabulated bulk p*K*_a_ clearly shows a deviation from the bulk behavior. The interface-sensitive spectroscopic data exhibit a pronounced shift of the equilibrium towards higher pH values in comparison to the equilibrium in the bulk, indicating an apparent shift of the p*K*_a_ at the liquid–vapor interface towards higher values. A similar deviation of the surface from the bulk p*K*_a_ was observed in SFG measurements on phenol solutions^17^ and UPS measurements of phenol aqueous nanoaerosols,^12^ where the signature of neutral phenol was still detectable at pH 12 and higher.

Werner and co-workers investigated shifts in the acid–base equilibria of carboxylic acids and alkyl amines at the liquid–vapor interface using liquid-jet XPS.^34^ They attributed the observed deviations of the apparent surface 
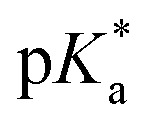
 from the bulk value to the different surface propensity of the acid and its conjugated base. To account for it they derived a modified description of the acid–base equilibrium, which takes the increased solubility and thus lower surface enhancement of the (charged and therefore more hydrated) conjugated organic species into account:^34^6
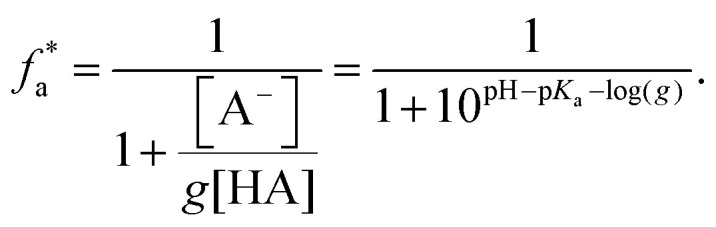


Here, *g* is defined as *g* := *g*_HA_/*g*_A^−^_ with *g*_HA_ and *g*_A^−^_ being surface enrichment factors of the conjugated acid and base, which relates their respective surface and bulk concentrations as [HA]^S^ = *g*_HA_[HA] and [A^−^]^S^ = *g*_A^−^_[A^−^], with [HA]^S^ and [A^−^]^S^ as the surface concentrations and [HA] and [A^−^] as the bulk concentrations. It follows from eqn (6) that the apparent surface 
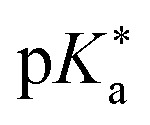
 can be defined as 
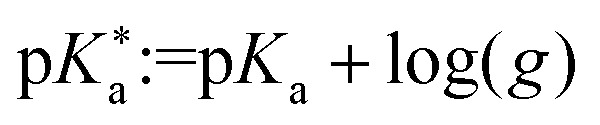
.

We used the surface concentrations *Γ* determined from the surface tension measurements to estimate the relative surface enrichment (*g* = *Γ*_PheoH_/*Γ*_PheO^−^_) of the two species to model the acid fraction of phenol according to eqn (6). The modified acid fraction 
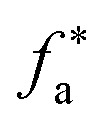
 is displayed as a red trace and agrees with the data from the photoemission measurements with an apparent surface 
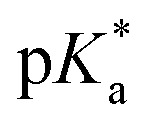
 of 11.1(3). This agreement suggests that the main contribution to the apparent shift is the relative surface enrichment of phenol *versus* phenolate in the narrow surface region probed in the PES experiment, rather than an actual shift in the acid–base equilibrium at the interface. This finding emphasizes the advantage of combining PES experiments with auxiliary surface tension measurements to cross-check or disentangle observations in the spectroscopic data, such as apparent shifts in acid–base equilibria at the liquid–vapor interface.

Fig. 4 also shows the dependence of the surface pressure *Π* = *γ*_0_ − *γ* on the pH (open circles) plotted against the right axis. The surface pressure follows the same sigmoid behavior associated with the bulk acid fraction and was therefore fitted with a modulated version of eqn (5) including a scaling factor, which was also used in ref. 39. Compared to the bulk equilibrium (blue trace) it is slightly shifted to an apparent 
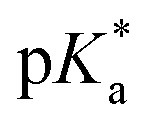
 of 10.17(2). The nature of this small deviation from the tabulated bulk p*K*_a_ is unclear. A potential reason might be the influence of the added electrolyte (NaOH) for the pH adjustment. Effects of the electrolyte concentration on interfacial pH shifts have been discussed in ref. 37.

### 3.4 Phenol and phenolate at the bulk p*K*_a_

In the previous section, we have discussed the relative abundance of phenol and phenolate as a function of pH for a fixed bulk concentration. In the present section, we will evaluate the adsorption isotherms of the two species at the nominal bulk p*K*_a_ of ∼10 as a function of bulk concentration.

Fig. 2(b) shows in addition to the data for the pure solutions at pH 5 (phenol) and pH 12 (phenolate) also the surface tension of phenol solution at pH 10 (grey circles), where both species coexist. A fit of the data at pH 10 using the Szyszkowski–Langmuir model (eqn (1)) yields Δ*G*_ads_ of −14.3(3) kJ mol^−1^. This value is closer to the one determined for phenol at pH 5 (Δ*G*_ads_ = −15.8(4) kJ mol^−1^) than to the one at pH 12 (Δ*G*_ads_ = −8.7(4) kJ mol^−1^), indicating that neutral species dominate the interface region. The Szyszkowski–Langmuir model, however, is typically used for binary solutions of one solute in a solvent, *e.g.*, phenol in water. Therefore, the individual surface-tension contributions in the present ternary system of phenol and phenolate in water cannot be easily disentangled. There are, however, predictive surface-tension models described in the literature which include the dissociation of organic molecules,^42^ though their application is not necessarily straightforward and beyond the scope of this work.

We have already shown, however, that photoelectron spectroscopy is capable of distinguishing the signals of phenol and phenolate, and based on these data we now determine the surface adsorption propensities of phenol and phenolate at the p*K*_a_, *i.e.*, pH ∼ 10. We performed measurements of phenol solutions at pH 10 as a function of the bulk concentration analogous to the measurements of pH 5 and 12 phenol solutions (see Section 3.2). When analyzing the valence spectra constraints were applied to the peak splitting, relative amplitudes, and widths based on the single-component spectra.

**Fig. 5:**
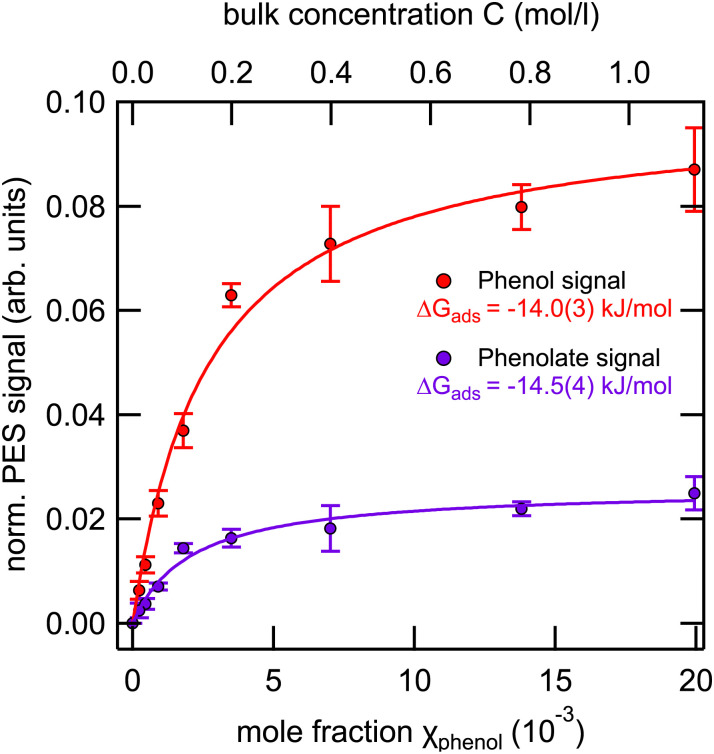
Integrated phenol (red) and phenolate (purple) peak intensities as a function of the mole fraction *χ* (bottom) and the bulk concentration *C* (top) for phenol solutions at pH 10, *i.e.*, at the bulk p*K*_a_. The shown error bars represent the standard deviation of the phenol valence signal due to stochastic errors.

Fig. 5 shows the integrated phenol (black symbols) and phenolate (red symbols) intensity, normalized to the signal of the 1b_1_ peak of pure water, as a function of the bulk mole fraction.

Applying the Langmuir adsorption model (eqn (3)) to the data we obtain values for Δ*G*_ads_ of −14.0(3) kJ mol^−1^ for phenol and −14.5(4) kJ mol^−1^ for phenolate. These values straddle the one obtained from surface tension measurements (−14.3(3) kJ mol^−1^), which combines the contributions from both species.

From these values it can be inferred that (i) the free energy of adsorption of the neutral phenol is slightly reduced compared to the pure solution at pH 5 [−16.1(1) kJ mol^−1^ to −14.0(3) kJ mol^−1^], and (ii) the free energy of adsorption of the negatively charged phenolate is noticeably increased compared to the pure solution at pH 12 [−8.6(3) kJ mol^−1^ to −14.5(4) kJ mol^−1^]. This behavior suggests the presence of cooperative effects between the two species, leading to a noticeable increase in the surface propensity of the phenolate in the presence of the neutral phenol. The nature of this cooperative effect, however, is unclear and would likely require computational support to be fully understood.

## 4 Conclusions

We have determined the surface propensity of phenol in aqueous solution and its acid–base equilibrium with phenolate. A detailed characterization of the composition of the liquid–vapor interface of aqueous organic solutions is a prerequisite to study heterogeneous processes at these interfaces. We employed liquid-jet valence and core-level photoelectron spectroscopy in conjunction with classical surface tension measurements using the Du Noüy–Padday method. While the two methods probe fundamentally different experimental quantities, the thermodynamic quantities determined from both methods, namely the free energies of adsorption Δ*G*_ads_ agree well with each other. The different values of Δ*G*_ads_ are summarized in Table 1 for different pH regimes.

The use of photoelectron spectroscopy enabled the simultaneous determination of Δ*G*_ads_ of the multi-component system, *i.e.*, phenol and phenolate, at the bulk p*K*_a_, which is elusive with surface tension measurements. Furthermore, we made use of the combination of the two techniques to investigate the acid–base equilibrium of phenol and phenolate. Photoelectron data alone would suggest a surface acid–base equilibrium deviating from the bulk equilibrium. However, taking into account the different surface concentrations determined from surface tensions of phenol and phenolate at the same bulk concentration, the equilibrium conditions for the bulk and the surface of the solution appear unchanged.

Throughout the literature, several studies can be found in which surface tension measurements are used for the qualitative interpretation of spectroscopic data, in particular photolectron spectroscopy and vibrational spectroscopies.^17,40,43–46^ However, the current study shows that a direct combination of spectroscopy with surface tension measurements supports a more robust investigation of liquid–vapor interfaces by allowing the cross-checking of experimental results of the two methods. In addition, the combination with surface tension measurements links photoelectron and vibrational spectroscopies to the existing vast literature of surface tension measurements, which have provided a wealth of information on the physicochemical properties of solution–vapor interfaces.

## Author contributions

CR conceived the project, analyzed the experimental data and wrote the manuscript with critical feedback from all co-authors. CR, RD, FT, TB, and DS performed the UPS measurements. CR, RD, TB, SG, HB, and RS performed the XPS measurements. CR, and LC performed surface tension measurements. BW, CN, and RS provided the endstations and support for the spectroscopic measurements. HB, and BW supervised the project.

## Data availability

Data for this article, including data displayed in Fig. 2–5 are available at our zenodo repository “Surface Accumulation and Acid–Base Equilibrium of Phenol at the Liquid–Vapor Interface” at https://doi.org/10.5281/zenodo.13235176.

## Conflicts of interest

There are no conflicts to declare.

## Supplementary Material

CP-026-D4CP02212B-s001
