# Supplementary material for: Surface accumulation and acid–base equilibrium of phenol at the liquid–vapor interface
Source: Phys Chem Chem Phys. 2024 Aug 23;26(43):27292–300. doi: 10.1039/d4cp02212b (PMC11348876; doi:10.1039/d4cp02212b)
Supplement: CP-026-D4CP02212B-s001 [file CP-026-D4CP02212B-s001.pdf]

Supplementary Information (SI) for Physical Chemistry Chemical Physics.
This journal is © the Owner Societies 2024

Electronic Supplementary Information to "Surface Accumulation and Acid-Base Equilibrium of Phenol at the Liquid-Vapor Interface"

Clemens Richter[1], Rémi Dupuy[2], Florian Trinter[1], Tillmann Buttersack[1], Louisa Cablitz[1], Shirin Gholami[1], Dominik Stemer[1], Christophe Nicolas[3], Robert Seidel[4], Bernd Winter[1], and Hendrik Bluhm[1]

[1]Fritz Haber Institute of the Max Planck Society, Faradayweg 4–6, 14195 Berlin, Germany
[2]CNRS, Laboratoire de Chimie Physique - Matière et Rayonnement, Sorbonne Université, F-75005 Paris Cedex 05, France
[3]Synchrotron SOLEIL, L'Orme des Merisiers, Saint-Aubin—BP 48 91192, Gif-sur-Yvette Cedex, France
[4]Helmholtz-Zentrum Berlin für Materialien und Energie, Hahn-Meitner-Platz 1, 14109 Berlin, Germany

Contents

1 Valence electron spectra of phenol aqueous solutions **2**
1.1 Single-component solutions at pH 5 and 12 2
1.2 Concentration-depedendent shifts in phenol valence states 3
1.3 Multi-component solutions at pH 10, i.e., the pK_a 3

2 Core-level spectra of phenol solutions **4**
2.1 Photoelectron angular distributions (PADs) 5

3 Normalization of phenol signal intensities **8**

4 Surface concentration Γ and surface fraction f_{Phenol} **9**

1 Valence electron spectra of phenol aqueous solutions

1.1 Single-component solutions at pH 5 and 12

Outer-valence photoelectron spectra were recorded for phenol solutions at pH 5, 10, and 12 over a wide concentration range using a helium plasma-discharge UV light source. The preferred photon energy was 40.814 eV (He II α radiation). Representative spectra and their decomposition are displayed in Fig. S1 for pH 5 and 12.

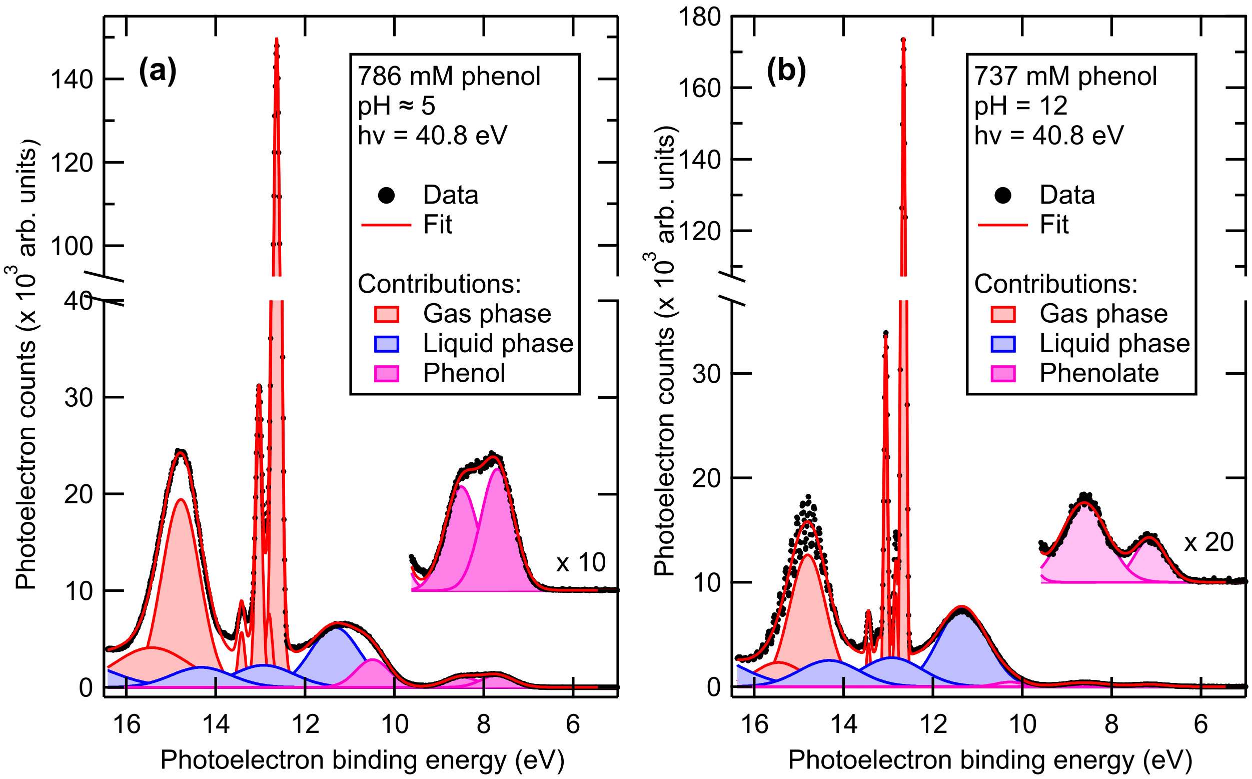

Figure S1: Fitted valence photoelectron spectra of phenol in aqueous solution at (a) pH 5 and (b) pH 12.

At pH 5 phenol is present purely in the neutral form, whereas at pH 12 it is predominately present as the dissociated negatively charged phenolate. Both spectra have been fitted with a complex fit model comprised by the sum of three distinct signal contributions: the prominent water gas phase (red peaks), the contributions of liquid water (blue peaks), and a contribution representing the phenol outer-valence states (violet peaks). High-resolution water gas-phase spectra, obtained by moving the liquid jet away from the light source, were used to determine the individual gas-phase peaks. For the subsequent use in the complex fit model the relative intensities and positions were constrained. "Liquid-only" spectra, in which the gas-phase contribution is smeared out by the application of a bias voltage of −30 V, were recorded to determine the individual liquid-phase peaks [10]. From these spectra the relative intensities and positions were used in the complex fit model. In addition to the pure water signatures, the signatures of the phenol outer-valence states were added to the complex fit based on the liquid-jet photoelectron spectra presented in Ref. [5] and on the phenol gas-phase spectra in Ref. [7]. Two peaks, the HOMO and HOMO-1 features, are well seperated at lower binding energies from the $1b_1$ state of liquid water. An additional auxiliary peak was required for a fully converged fit. We attribute this auxiliary peak to the onset of the other phenol valence states. As the gas-phase reference spectra tabulated in Ref. [7] indicate, these states strongly overlap with the water valence states. With increasing phenol concentration it is necessary to account for the phenol spectral contributions as they visibly contribute to the intensity in the region of the $1b_1$ state of liquid water.

1.2 Concentration-depedendent shifts in phenol valence states

While analyzing the valence electron spectra we noticed shifts in the phenol valence peak positions as a function of the concentration. These shifts are displayed in Fig. S2(a) as the apparent binding energy of the HOMO (open symbols) and HOMO-1 (filled symbols) orbitals relative to the $1b_1$ orbital of liquid water as a function of the surface concentration.

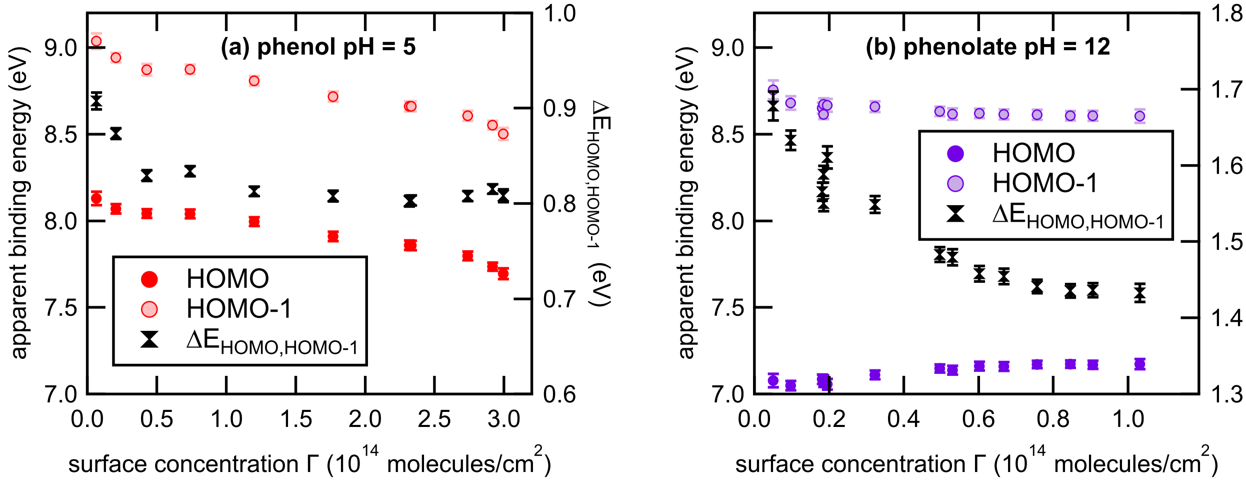

Figure S2: Binding energy of the HOMO (open symbols) and HOMO-1 (closed symbols) orbitals with respect to the $1b_1$ state of liquid water as a function of the phenol (a) and phenolate (b) surface concentration Γ. The black symbols represent the splitting between HOMO and HOMO-1 ($\Delta E_{HOMO,HOMO-1}$, right axis) as a function of Γ. The error bars were determined from standard deviations of multiple UPS spectra. They are of the size of the symbols.

With increasing surface (and hence also bulk) concentration the HOMO and HOMO-1 states shift towards lower binding energies by 0.4-0.5 eV. Similar shifts have been observed for the I 5p valence electron signal of tetrabutylammonium iodide (TBAI) solutions as a function of the bulk concentration [1].

Figure S2(a) also shows the HOMO/HOMO-1 splitting $\Delta E_{\mathrm{HOMO,HOMO-1}}$, plotted against the right axis. The splitting decreases from 0.9 eV to 0.8 eV in the range of low surface concentrations and subsequently stays constant for surface concentrations above 10^{14} molecules/cm^2. The nature of these shifts is as of yet unclear; elucidation of the underlying mechanism would likely require measurements of absolute binding energies and work functions in connection with computational approaches. These experiments, however, are outside of the scope of this work.

As already shown for phenol, the position of the HOMO and HOMO-1 orbitals was analyzed also for phenolate solutions. Figure S2(b) shows the evolution of the HOMO (dark violet symbols) and HOMO-1 (light violet symbols) as a function of the surface concentration.

The depicted range of the surface concentrations corresponds to bulk concentrations ranging from 50 mM to 2500 mM. Black symbols correspond to the binding-energy splitting between the HOMO and HOMO-1 levels ($\Delta E_{\mathrm{HOMO,HOMO-1}}$). While both the HOMO and HOMO-1 levels each show less variation compared to the corresponding valence levels of the neutral phenol, the difference between the two shows a larger change as a function of surface concentration. This is caused by the fact that the two levels approach each other with increasing surface coverage in the case of phenolate, while the larger individual shifts in the case of phenol are in the same direction.

1.3 Multi-component solutions at pH 10, i.e., the pK_a

Figure S3 shows the valence electron spectrum of a phenol solution at the pK_a, i.e., pH ∼10.

Under these conditions both species, the neutral phenol and the negatively charged phenolate, are present at equal concentrations in the bulk solution. The fit model for these spectra is constructed analogous to the model for the single-solute spectra (see Fig. S1), i.e., it contains the contributions of gaseous and liquid water plus the solute contribution. In this case, the solute contribution consists of the sum of the HOMO and HOMO-1

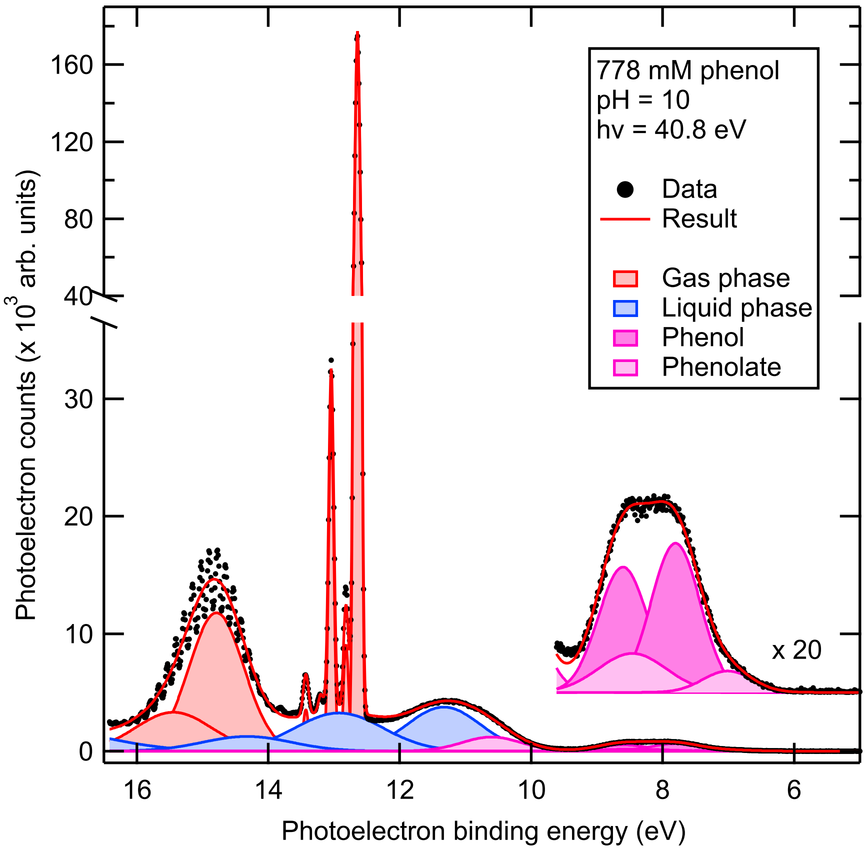

Figure S3: Fitted valence photoelectron spectrum of phenol in aqueous solution at pH 10, i.e., at the bulk pK_a.

signatures of phenol and phenolate and a shared auxiliary peak overlapping with the $1b_1$ feature of liquid water. In all valence electron spectra at pH 10, the different signal contributions of phenol and phenolate are clearly noticeable. However, due to their strong overlap fit constraints have been used to ensure consistent convergence of the fit. The constraints were applied to the widths, amplitudes, and energy splitting of the HOMO and HOMO-1 features based on the respective single-solute spectra.

2 Core-level spectra of phenol solutions

To get a comprehensive picture of the surfactant properties of phenol in aqueous environments, core-level photoelectron spectra of phenol solutions at pH 5, 10, and 12 were recorded as a function of the bulk concentration at the SOLEIL synchrotron facility (Paris, France). Representative C 1s core-level spectra for these three samples are displayed in Fig. S3 in the electronic supplementary material.

Analogous to the valence electron spectra (Fig. S1), the fit model for the C 1s spectra was derived for the single-solute spectra at pH 5 and 12, i.e., the neutral phenol and the negatively charged phenolate. The fit consisted of two Gaussian peaks on a linear background. The two peaks are assigned to the aliphatic carbon (CH_x) at higher kinetic energy and the hydroxylic carbon (COH / CO^-) at lower kinetic energy. For spectra at pH 5 and concentrations exceeding 200 mM a small contribution of phenol vapor had to be included for satisfactory fit results. This was justified due to the significant vapor pressure of solid phenol of about 0.5 mbar at room temperature. Additional phenol gas-phase spectra were recorded to characterize the gas-phase signatures, specifically the peak splitting and relative amplitudes, to be used in the fitting of the phenol solution spectra. C 1s spectra containing more than one species, i.e., phenol vapor and/or phenol and phenolate at pH 10, were fitted using constraints on the widths, amplitudes, and peak splitting determined from the respective single-component spectra.

The O 1s spectra were fitted using Voigt profiles for the significant contribution of the water vapor phase (sharp signal at low kinetic energies). The vapor signal showed a pronounced asymmetry requiring multiple

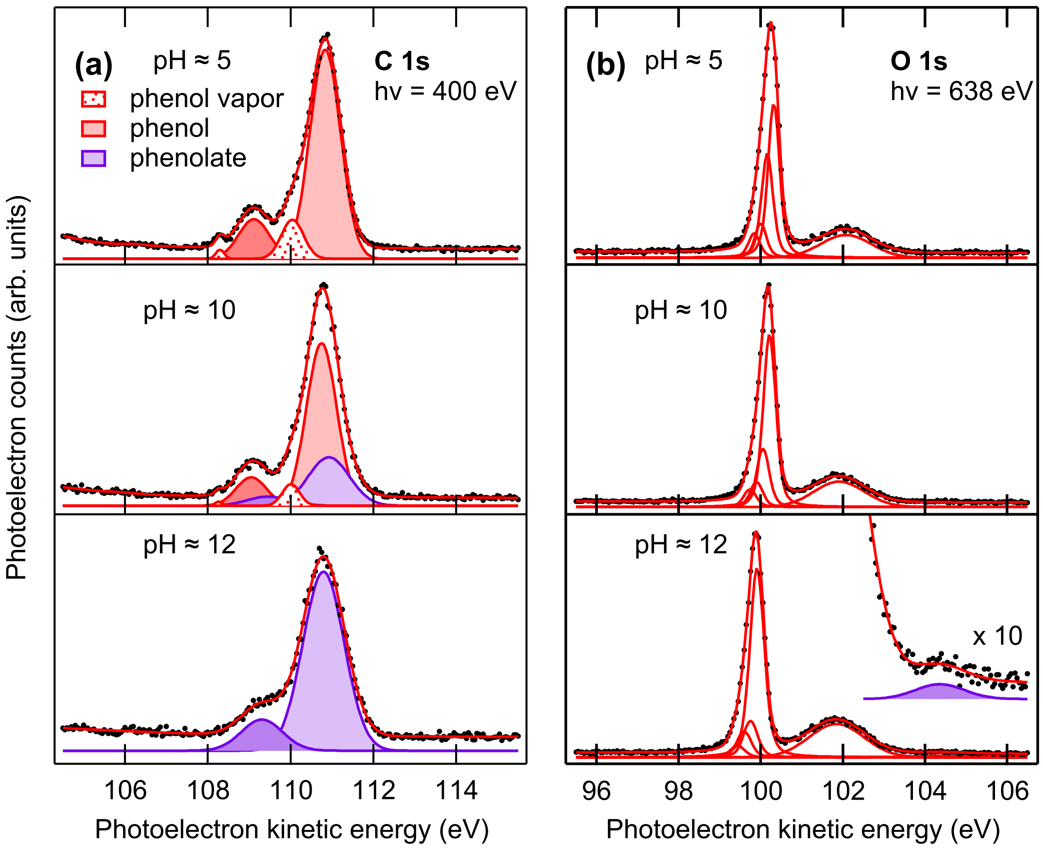

Figure S4: C 1s and O 1s core-level spectra of 800 mM phenol solutions at pH 5, 10, and 12. Recorded with $h\nu(\text{C 1s}) = 400\,\text{eV}$ and $h\nu(\text{O 1s}) = 640\,\text{eV}$.

equidistant Voigt profiles to account for the vibrational contributions to the gas-phase signal. The liquid-phase contribution was fitted using Gaussian peaks. A solute signal contribution was only visible as an additional peak at approximately 104 eV kinetic energy at high phenolate (pH 12) concentrations exceeding 800 mM (see inset in Fig. S4(b)).

The phenol C 1s spectra are comprised of two peaks: the CH_x carbons of the main ring structure and the COH carbon at lower binding energy. The neutral phenol and the negatively charged phenolate can be distinguished by the splitting of the two peaks which is 1.66(3) eV for phenol and 1.45(2) eV for phenolate. Analogous to the UPS investigations, XPS measurements were performed to determine the surface adsorption of phenol from the core-level spectra. From these measurements we obtain a ΔG_{ads} value of –16.7(3) kJ/mol for the neutral phenol and –8.1(2) kJ/mol for the phenolate. These values are in good agreement with the results from the valence electron spectra and the surface tension measurements. All values are summarized in Table 2.

The free energies of adsorption determined from core-level spectra at pH 10 are –12.7(6) kJ/mol for phenol and –13.7(4) kJ/mol for phenolate. These values are lower than the ones from the valence spectra with the phenolate exhibiting a slightly higher value for ΔG_{ads}. We believe that this behavior can be ascribed to the relatively strong spectral overlap of the two species in the C 1s spectra at pH 10, which generally complicates a quantitative analysis of the core-level spectra when the two species are present at the same time. However, the same trends of a lowered adsorption energy for phenol and noticeably enhanced adsorption energy for phenolate are also observed in the XPS measurements.

2.1 Photoelectron angular distributions (PADs)

To obtain additional information on the relative surface propensity of phenol and phenolate we also performed measurements of the C 1s photoelectron angular distributions (PADs) to test whether changes in the surface concentration correlate with relative changes of the orientation of the phenol molecule at the liquid–vapor

interface.

A detailed description of these types of measurements is given in Refs. [3, 4, 2]. In brief, C 1s spectra of phenol solutions, at pH 5 and 12 at different bulk and hence different surface concentrations, were measured at varying angles between the direction of the linear light polarization and the electron detection. The incident photon energy was 400 eV.

The measurements of C 1s PADs were mainly performed using linearly polarized synchrotron radiation from the PLEIADES beamline. Two sets of PAD measurements were recorded at the UE52_SGM beamline at the BESSY II synchrotron facility. The C 1s spectra of phenol solutions at pH 5 and pH 12 were recorded at low and high bulk concentrations. Figure S5(a) shows a representative data set of these PAD measurements for 50 mM phenol solution at pH 5.

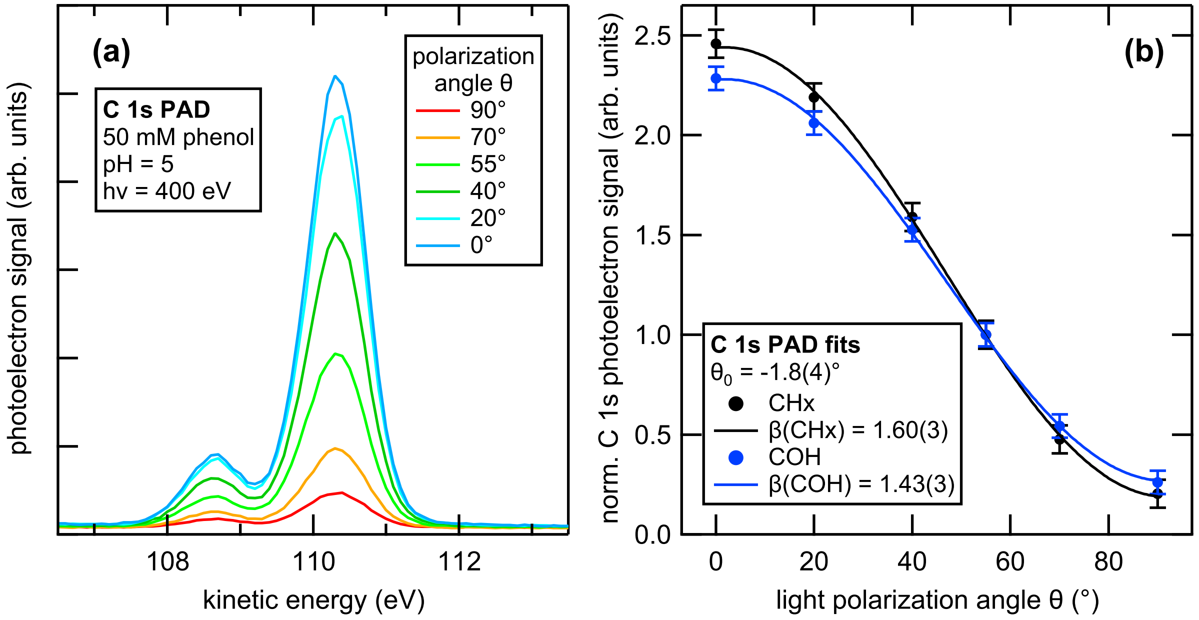

Figure S5: (a) C 1s PAD measurements of pH 5 phenol solutions at 50 mM concentration and 400 eV photon energy. (b) C 1s intensities of the CH_x and COH carbons as a function of the light polarization angle θ. Intensities were normalized to the photon flux at each polarization angle.

The spectra are shown as measured. From the fitted C 1s spectra the intensities of the hydroxyl and aliphatic carbons were determined and normalized to the photon flux at the different polarization angles. These intensities are shown in Fig. S5(b). They were fitted using the model described in Refs. [3, 4, 2] to determine the anisotropy parameters β for the different carbon species. All C 1s anisotropy parameters determined from phenol solutions during different synchrotron beamtimes are compiled in Table 1.

The first row in Table 1 shows the β parameter for phenol vapor, which was used as a reference to determine the reduced anisotropy parameter R_β (see main text for details).

Analogous measurements of phenol vapor were performed to assess the degree of intramolecular scattering at the different carbon species in phenol, namely COH and CH_x. The anisotropy parameters β were determined using the established fit models [3, 4, 2]. Subsequently, the reduced β parameters $R_\beta = R_{liq}/R_{gas}$ were determined, which account for the nascent elastic scattering properties of the molecule. Figure S6 shows the resulting R_β values for the different carbon signals of phenol and phenolate as a function of the surface concentration Γ.

The uncertainties in R_β were computed by propagating the errors in β_{gas} and β_{liq}, which were determined from the fit results. The main source of experimental uncertainty in these experiments are changes in the relative alignment of the liquid jet and the synchrotron beam in front of the entrance of the electron analyzer during the measurements.

The β values for phenol and phenolate in water are noticeably smaller than the one for phenol vapor due to the elastic scattering of the C 1s photoelectrons by the surrounding water. For the pure phenol species (at pH 5), $R_\beta(CH_x)$ and $R_\beta(COH)$ are comparable for low surface concentration, indicating that both parts of the molecule are solvated equally (within the experimental uncertainty). With increasing concentration the

Table 1: Compilation of C 1s β parameters for phenol / phenolate aqueous solutions determined from PAD measurements at 400 eV photon energy. Concentrations C are given in mM and surface concentrations Γ are given in 10^{14} molecules/cm^2.

**Campaign**	**pH**	**C**	Γ	f_{Phenol}	$\beta_{\mathrm{CH_x}}$	β_{COH}	$\mathrm{R}_{\beta_{\mathrm{CH_x}}}$	$\mathrm{R}_{\beta_{\mathrm{COH}}}$
SOLEIL 2021	-	-	-	-	1.85(5)	1.74(7)	1	1
SOLEIL 2021	5	400	2.8	0.78	1.54(2)	1.35(3)	0.83(4)	0.78(5)
BESSY II 2021	5	28	0.8	0.21	1.55(4)	1.43(4)	0.84(4)	0.82(6)
BESSY II 2021	5	305	2.7	0.74	1.54(2)	1.35(4)	0.84(4)	0.77(5)
SOLEIL 2023	5	50	1.2	0.32	1.60(3)	1.43(3)	0.87(4)	0.82(5)
SOLEIL 2023	5	400	2.8	0.78	1.68(5)	1.35(3)	0.91(5)	0.77(5)
SOLEIL 2023	12	200	0.2	0.05	1.51(3)	1.27(5)	0.82(4)	0.73(6)
SOLEIL 2023	12	1000	0.7	0.18	1.55(4)	1.30(7)	0.84(5)	0.74(7)

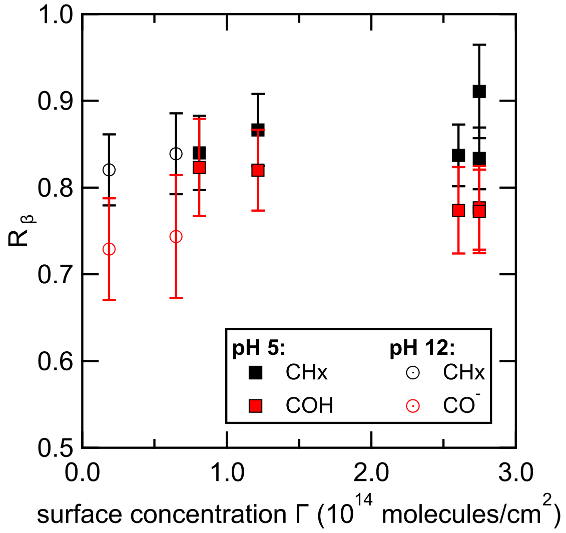

Figure S6: R_β values for the C 1s signal of neutral phenol (closed symbols) and negatively charged phenolate (open symbols) solutions as a function of the estimated surface concentration Γ. The black symbols correspond to the $\mathrm{CH_x}$ carbon and the red symbols to the COH/$\mathrm{CO^-}$ carbon.

values of R_β diverge from each other with $\mathrm{R}_\beta(\mathrm{CH_x})$ increasing and R_β(COH) decreasing, which clearly indicates a favorable orientation of the molecule with the hydroxyl carbon pointing towards the solution bulk and the hydrophobic ring carbons pointing outwards at the liquid–vapor interface. This favorable orientation is in line with chemical intuition and with observations in previous experiments using non-linear spectroscopy, namely SHG and SFG [6, 8]. Possible changes in the surface structure or composition at high surface concentrations, as suggested by the authors of Ref. [6], cannot be unambiguously corroborated with the current data set and would require a more detailed set of PAD measurements which are outside the scope of this work.

For the negatively charged phenolate solutions $\mathrm{R}_\beta(\mathrm{CO^-})$ is noticeably smaller, clearly indicating a preferential orientation of the molecule with the polar end towards the solution, even at low surface concentration, again in accordance with chemical intuition.

3 Normalization of phenol signal intensities

From the phenol valence electron spectra and the core-level spectra at different bulk concentration the Gibbs free energies of adsorption ΔG_{ads} for the different phenol species were determined. For this purpose the integrity of each spectrum wihtin a data set, such as the concentration-dependent phenol signal intensity at a fixed solution pH, needed to be validated. In case of data recorded with the helium plasma-discharge lamp, reference spectra of pure water (including 25 mM NaCl for sample conductivity) were regularly taken before and after measuring the phenol solutions. The congruence of the reference spectra ensured the stability of the liquid-jet position in front of the analyzer entrance aperture. In addition, referencing the phenol valence signal intensity at concentration C ($I_{phenol,C}$) to the intensity of the liquid $1b_1$ state of the reference spectra ($I_{1b_1,C=0}$) enabled the comparison of phenol spectra recorded at different days, i.e., at varying alignment of the liquid jet:

$$I'_{phenol,C} = \frac{I_{phenol,C}}{I_{1b_1,C=0}}.$$

This treatment is conceptually similar to the surface tension measurements of the phenol solutions in reference to the surface tension of pure water. The results of this procedure are shown in Fig. S7(a).

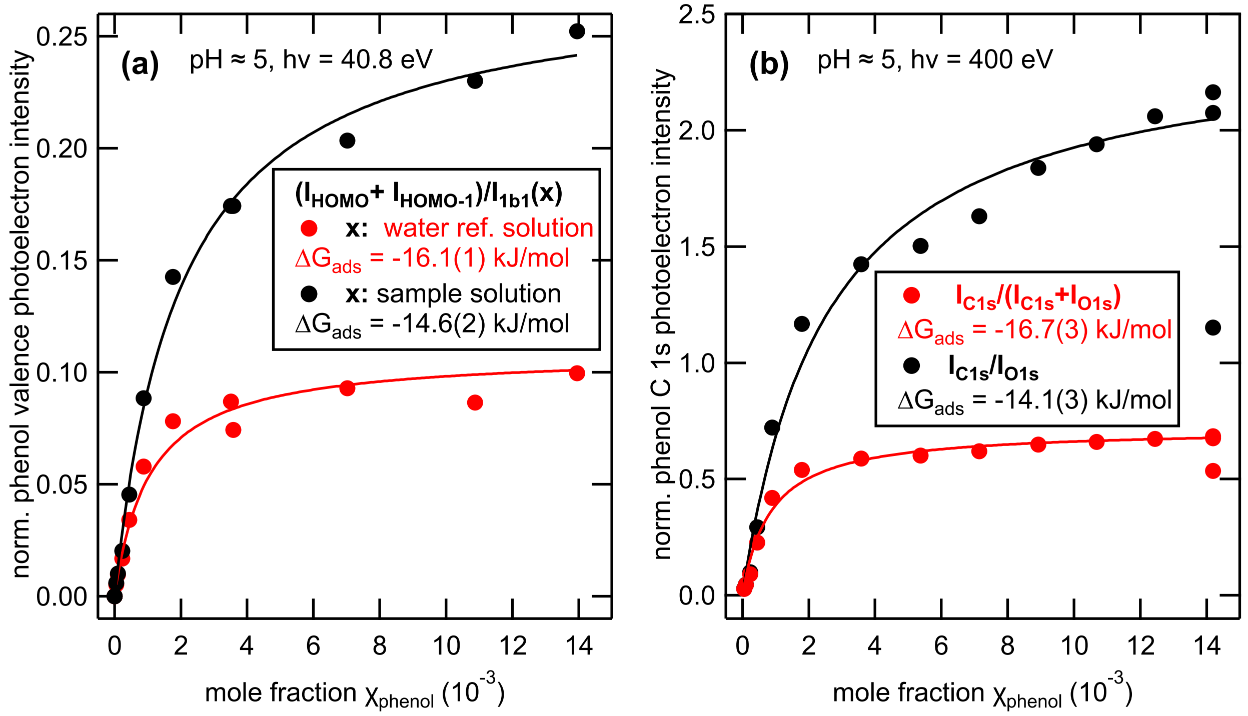

Figure S7: Different normalization approached for the concentration dependence of the normalized phenol photoelectron signals. (a) Black symbols show phenol valence-state intensities normalized to the liquid $1b_1$ state for the respective sample solution. Red symbols show the phenol valence-state intensities normalized to the liquid $1b_1$ state of the pure water reference solution. (b) Black symbols show the phenol C 1s intensity normalized to the O 1s intensity for the respective sample solution. Red symbols show the phenol C 1s intensity normalized to the the sum of C 1s and O 1s intensities for the respective sample solution. The data was fitted (solid curves) using a Langmuir adsorption model [11].

In case of the core-level spectra (see Fig. S7(b)), a different approach had to be adjusted due to an enhanced sensitivity of the jet alignment at the PLÉIADES setup. Compared to the relatively broad XUV beam of the helium plasma-discharge lamp, the focus size of the synchrotron beam is on the order of the liquid jet (tens of μm). This resulted in a greater likelihood of changes in the overlap between the liquid jet and the synchrotron beam upon switching between sample solutions. To address this higher degree of setup-specific sensitivity a O 1s–C 1s–O 1s measurement scheme was applied for each phenol solution. Congruence of the O 1s spectra validated the signal stability for each phenol solution. However, the direct comparison with a pure water reference solution

was not possible. Instead the phenol C 1s signal of each solution at concentration C [$I_C(C\ 1s)$] was normalized by the sum of the C 1s and O 1s intensity of the solution [$I_C(C\ 1s) + I_C(O\ 1s)$]:

$$I_C(C\ 1s)' = \frac{I_C(C\ 1s)}{I_C(C\ 1s) + I_C(O\ 1s)}.$$

This approach has previously been applied to acetonitrile-water mixtures [9]. By using this normalization the additional effect of electron signal attenuation by the build-up of a surface-active carbon layer, which is present in the O 1s signal, is minimized.

In the data analysis, we observed that the normalization of the solute signal intensity has a significant impact on the determination of the Gibbs free energy of adsorption ΔG_{ads} using the Langmuir adsorption model [11]. When the phenol intensity was not referenced / normalized as outlined above in order to compensate the attenuation of liquid-water signal ($1b_1$ or O 1s) by the surface enrichment of the phenol, the values of ΔG_{ads} were consistently too low compared to the ones obtained in surface tension measurements. The values of ΔG_{ads} determined from the different measurements (UPS, XPS, and surface tension measurements) are compiled in Table 2.

Table 2: Compilation of Gibbs free energies of adsorption ΔG_{ads} for phenol / phenolate aqueous solutions determined with UPS, XPS, and surface tension measurements. All values are given in kJ/mol.

<table>
<tr><th>Species</th><th>pH</th><th>$\Delta \mathbf{G}_{ads}^{UPS}$</th><th>$\Delta \mathbf{G}_{ads}^{XPS}$</th><th>$\Delta \mathbf{G}_{ads}^{ST}$</th></tr>
<tr><td>phenol</td><td>5</td><td>–16.1(1)</td><td>-16.7(3)</td><td>–15.8(4)</td></tr>
<tr><td>phenolate</td><td>12</td><td>–8.6(3)</td><td>–8.1(2)</td><td>–8.5(3)</td></tr>
<tr><td>phenol</td><td>10</td><td>–14.0(3)</td><td>–12.7(6)</td><td rowspan="2">-14.3(3)</td></tr>
<tr><td>phenolate</td><td>10</td><td>–14.5(4)</td><td>–13.7(4)</td></tr>
</table>

The direct comparison of ΔG_{ads}^{UPS} and ΔG_{ads}^{XPS} indicates that both normalization approaches deliver similar results. Furthermore, the results obtained by photoelectron spectroscopy and surface tension measurements show a very good agreement thus increasing the confidence in the applied approach to reference / normalize the spectroscopic intensities.

4 Surface concentration Γ and surface fraction f_{Phenol}

The number of phenol molecules at the surface n_{phenol} of pure phenol with the dimension of 1/cm^2 can be calculated from its density ρ (1.07 g/cm^3), the molar mass M (94.11 g/mol), and the Avogadro number N_A:

$$n_{\text{Phenol}} = \left(\frac{\rho \cdot N_A}{M}\right)^{2/3} = 3.6 \cdot 10^{14} \text{ molecules / cm}^{\mathbf{2}}. \quad (1)$$

Based on the simplified assumption that the structural ordering of phenol molecules in pure phenol is similar to the one at the water–phenol interface, we now estimate the fraction of a monolayer at the aqueous–vapor interface by:

$$f_{\text{Phenol}} = \frac{\Gamma}{n_{\text{Phenol}}}. \quad (2)$$

This means that for $\Gamma = 3 \cdot 10^{14}$ molecules per cm^2 (the maximum determined value from surface tension measurements for a 800 mM solution) a fraction of about 0.87 of a monolayer is formed. In other words, under these conditions the water interface is almost completely covered with phenol molecules.

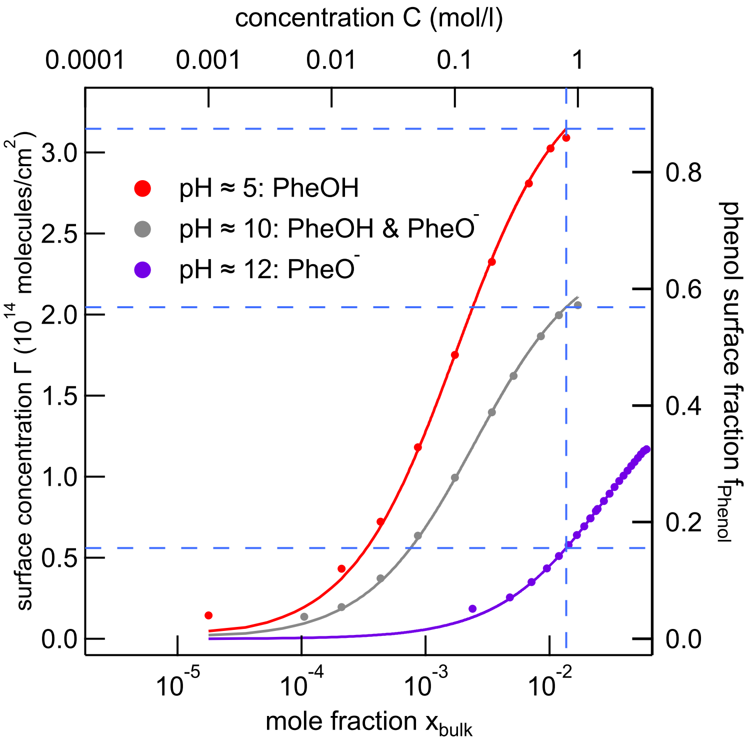

Figure S8: Surface concentration Γ and maximum surface fraction f_{Phenol} of phenol solutions at pH 5, 10, and 12.

References

[1] B. Credidio, M. Pugini, S. Malerz, F. Trinter, U. Hergenhahn, I. Wilkinson, S. Thürmer, and B. Winter. Quantitative electronic structure and work-function changes of liquid water induced by solute. *Phys Chem Chem Phys*, 24(3):1310–1325, 2022.

[2] R. Dupuy, J. Filser, C. Richter, T. Buttersack, F. Trinter, S. Gholami, R. Seidel, C. Nicolas, J. Bozek, D. Egger, H. Oberhofer, S. Thürmer, U. Hergenhahn, K. Reuter, B. Winter, and H. Bluhm. Ångstrom-depth resolution with chemical specificity at the liquid-vapor interface. *Phys Rev Lett*, 130(15):156901, apr 2023.

[3] R. Dupuy, J. Filser, C. Richter, R. Seidel, F. Trinter, T. Buttersack, C. Nicolas, J. Bozek, U. Hergenhahn, H. Oberhofer, B. Winter, K. Reuter, and H. Bluhm. Photoelectron angular distributions as sensitive probes of surfactant layer structure at the liquid–vapor interface. *Phys Chem Chem Phys*, 24(8):4796–4808, 2022.

[4] R. Dupuy, S. Thürmer, C. Richter, T. Buttersack, F. Trinter, B. Winter, and H. Bluhm. Core-level photoelectron angular distributions at the liquid–vapor interface. *Accounts Chem Res*, 56(3):215–223, jan 2023.

[5] D. Ghosh, A. Roy, R. Seidel, B. Winter, S. Bradforth, and A. I. Krylov. First-Principle Protocol for Calculating Ionization Energies and Redox Potentials of Solvated Molecules and Ions: Theory and Application to Aqueous Phenol and Phenolate. *J Phys Chem B*, 116(24):7269–7280, jun 2012.

[6] J. M. Hicks, K. Kemnitz, K. B. Eisenthal, and T. F. Heinz. Studies of liquid surfaces by second harmonic generation. *J. Phys. Chem.*, 90(4):560–562, 1986.

[7] K. Kimura. *Handbook of HeI photoelectron spectra of fundamental organic molecules: ionization energies, ab initio assignments, and valence electronic structure for 200 molecules.* Japan Scientific Societies Press, 1981.

[8] R. Kusaka, T. Ishiyama, S. Nihonyanagi, A. Morita, and T. Tahara. Structure at the air/water interface in the presence of phenol: A study using heterodyne-detected vibrational sum frequency generation and molecular dynamics simulation. *Phys Chem Chem Phys*, 20(5):3002–3009, 2018.

[9] K. A. Perrine, M. H. C. Van Spyk, A. M. Margarella, B. Winter, M. Faubel, H. Bluhm, and J. C. Hemminger. Characterization of the Acetonitrile Aqueous Solution/Vapor Interface by Liquid-Jet X-ray Photoelectron Spectroscopy. *J. Phys. Chem. C*, 118(50):29378–29388, Dec. 2014.

[10] S. Thürmer, S. Malerz, F. Trinter, U. Hergenhahn, C. Lee, D. M. Neumark, G. Meijer, B. Winter, and I. Wilkinson. Accurate vertical ionization energy and work function determinations of liquid water and aqueous solutions. *Chem Sci*, 12(31):10558–10582, 2021.

[11] J. Werner, I. Persson, O. Björneholm, D. Kawecki, C.-M. Saak, M.-M. Walz, V. Ekholm, I. Unger, C. Valtl, C. Caleman, G. Öhrwall, and N. L. Prisle. Shifted equilibria of organic acids and bases in the aqueous surface region. *Phys Chem Chem Phys*, 20(36):23281–23293, 2018.
